# Chronic-Recurrent, Limb-Predominant Grover Disease in a Young Adult: A Clinicopathological Diagnostic Challenge

**DOI:** 10.3390/diagnostics16193176

**Published:** 2026-09-30

**Authors:** Laura Maghiar, Andreea-Adriana Neamțu, Teodor-Andrei Maghiar, Mariana Ganea, Ovidiu Țică, Ruxandra Florina Bodog, Teodora Maria Bodog, Octavia Gligor, Laura Grațiela Vicaș, Mădălin Florin Ganea, Csaba Nagy, Ilarie Brihan

**Affiliations:** 1 Department of Psycho-Neurosciences and Rehabilitation, Faculty of Medicine and Pharmacy, University of Oradea, Universității Street, No. 1, 410087 Oradea, Romania; laura.maghiar@uoradea.ro (L.M.); ibrihan@uoradea.ro (I.B.); 2 Faculty of Medicine and Pharmacy, University of Oradea, 1st December Square, No. 10, 410028 Oradea, Romania; ovidiu.tica@gmail.com (O.Ț.); bodog.ruxandraflorina@student.uoradea.ro (R.F.B.); bodog.teodoramaria@student.uoradea.ro (T.M.B.); ganea.madalinflorin@student.uoradea.ro (M.F.G.); 3 Bihor County Emergency Clinical Hospital, 410169 Oradea, Romania; 4 Department of Toxicology, “Victor Babeș” University of Medicine and Pharmacy, Eftimie Murgu Square, No. 2, 300041 Timișoara, Romania; andreea.neamtu@umft.ro; 5 Research Centre for Pharmaco-Toxicological Evaluation, “Victor Babeș” University of Medicine and Pharmacy, Eftimie Murgu Square, No. 2, 300041 Timișoara, Romania; 6 Department of Pathology, Clinical County Emergency Hospital of Arad, Andrenyi Karoly Street, No. 2–4, 310037 Arad, Romania; 7 Department of Pathology, “Pius Brînzeu” Clinical County Emergency Hospital Timișoara, Liviu Rebreanu Boulevard, No. 156, 300723 Timișoara, Romania; 8 Department of Surgery, Faculty of Medicine and Pharmacy, University of Oradea, Universității Street, No. 1, 410087 Oradea, Romania; 9 Department of Surgery, Pelican Hospital, Corneliu Coposu Street, No. 2, 410450 Oradea, Romania; 10 Pharmacy Department, Faculty of Medicine and Pharmacy, University of Oradea, 1st December Square, No. 10, 410028 Oradea, Romania; lvicas@uoradea.ro; 11 Department of Preclinical Disciplines, Faculty of Medicine and Pharmacy, University of Oradea, 1st December Square, No. 10, 410028 Oradea, Romania; octavia.gligor@uoradea.ro; 12 Doctoral School of Biomedical Sciences, University of Oradea, Universității Street, No. 1, 410087 Oradea, Romania; nagycsaba95@yahoo.com

**Keywords:** Grover disease, transient acantholytic dermatosis, acantholysis, clinicopathological correlation, histopathology, direct immunofluorescence, dermoscopy, young adult, acitretin, serum IgE

## Abstract

Grover disease (GD), or transient acantholytic dermatosis, is an acquired acantholytic dermatosis that classically affects the trunk of older men, although persistent, recurrent and atypically distributed forms are increasingly recognised. We report a 29-year-old man with chronic-recurrent, intensely pruritic GD, with an estimated onset approximately six months before his first documented dermatologic assessment. The eruption predominantly involved the upper and lower limbs, with limited truncal involvement. Because of the atypical age and distribution, and because acantholysis was focal and minimal in individual specimens, the diagnosis rested on clinicopathological correlation across serial biopsies obtained during separate disease episodes, which showed spongiotic and acanthotic changes, focal parakeratosis, lichenoid and perivascular inflammation, and focal acantholysis with dyskeratosis. Direct immunofluorescence, performed during topical corticosteroid treatment, was nonspecific and did not support an autoimmune blistering disorder, although this result must be interpreted with caution. Laboratory investigations were unremarkable apart from a moderately elevated total serum immunoglobulin E (IgE) (223.2 IU/mL), a nonspecific finding that does not establish a type 2 immune phenotype in this patient. A combined regimen of acitretin, an oral antihistamine, a topical corticosteroid and barrier-directed care was temporally associated with marked improvement, the Dermatology Life Quality Index falling from 17/30 to 5/30 over three months. We briefly review the histopathological patterns, differential diagnosis and management of GD and its emerging molecular and immunological context. This case illustrates the breadth of the clinical spectrum of GD, the diagnostic pitfalls of focal acantholysis and of immunofluorescence performed under treatment, and the need to consider GD in young patients with chronic-recurrent, predominantly extra-truncal eruptions.

## 1. Introduction

Grover disease (GD), also termed transient acantholytic dermatosis, is an acquired acantholytic dermatosis characterised by pruritic papules and papulovesicles that classically affects the trunk of fair-skinned men older than 40–50 years and is precipitated by heat, sweating, xerosis, occlusion, ultraviolet exposure, prolonged bed rest, or drugs [1]. Although its name implies a self-limited course, persistent and chronic-recurrent forms are well recognised, and GD is now best understood as a histologic reaction pattern in which several pathways converge on focal acantholytic dyskeratosis [1,2]. Recent work has linked GD to somatic ATP2A2 variants [3] and to extracellular signal-regulated kinase (ERK) hyperactivation in keratinocytes [4], and has described a type 2 immune signature in lesional skin [5]; these findings are summarised below as context, but none of them was investigated in the present patient. Presentation in young adults, and a distribution that spares the trunk in favour of the limbs, are both uncommon and may delay diagnosis, particularly when acantholysis is focal or minimal in individual biopsy specimens. The contribution of this report is therefore not the description of a new phenotype but the documentation of the diagnostic process in such a patient. We describe how serial biopsies obtained during separate disease episodes, direct immunofluorescence and clinicopathological correlation supported the diagnosis of chronic-recurrent, limb-predominant GD in a 29-year-old man, and we discuss the pitfalls encountered, including treatment-related histological modification and the limited value of immunofluorescence performed under topical corticosteroid therapy.

## 2. Case Presentation

A 29-year-old man, a law-enforcement officer whose occupation entailed intense physical activity and frequent heat exposure, was evaluated for recurrent, intensely pruritic papular eruptions. He was first assessed at another hospital and was subsequently referred to the dermatology service of a tertiary emergency hospital, where the diagnostic work-up and treatment described below were carried out. He was a non-smoker, denied drug allergies and reported no exposure to medications known to trigger GD. His personal and family medical history was otherwise unremarkable; in particular, there was no personal history of atopic dermatitis and no family history of Darier or Hailey–Hailey disease. Written informed consent was obtained for publication of clinical data and images.

The exact date of disease onset could not be established retrospectively. At the first documented dermatologic assessment, hereafter designated month 0, the patient reported that the eruption had been present for approximately six months (estimated onset at month −6) and had followed a recurrent course, with periods of improvement and subsequent exacerbation. Intensely pruritic erythematous papules predominantly involving the extremities were documented. In view of the young age of the patient and the extra-truncal distribution, the clinical differential diagnosis at that time included papular eczema, folliculitis, an arthropod reaction and scabies, and punch biopsies were obtained from the upper limb, one from the arm and one from the forearm. Histopathological examination showed hyperkeratosis, focal attenuation of the granular layer, mild acanthosis and spongiosis, vacuolar change of basal keratinocytes, focal dyskeratosis and minimal suprabasal acantholysis, together with a superficial polymorphous inflammatory infiltrate with a perivascular and peri-adnexal distribution. The combination of focal suprabasal acantholysis with dyskeratosis, in the absence of the extensive acantholysis of the hereditary acantholytic dermatoses and of pemphigus, was considered compatible with transient acantholytic dermatosis (GD) in the clinical context.

At follow-up two months later (month 2), the diagnosis of GD was considered clinically and histopathologically supported. Pruritus remained intermittent and of moderate intensity. Treatment at that time consisted of bilastine 20 mg/day together with topical and barrier-directed therapy; the patient was also taking oral vitamin D and zinc supplementation, which had been prescribed by another physician before referral to our service and for which no indication was documented in the records available to us. Over the following months the disease remained intermittently symptomatic under this regimen. At month 9, the patient presented with a clinically significant recurrent flare, characterised by a predominantly limb-based papular eruption and substantial impairment of quality of life, with a Dermatology Life Quality Index (DLQI) score of 17/30. This flare prompted repeat histopathological assessment and initiation of systemic retinoid therapy; the overall course is summarised in Table 1.

**Figure 1:**
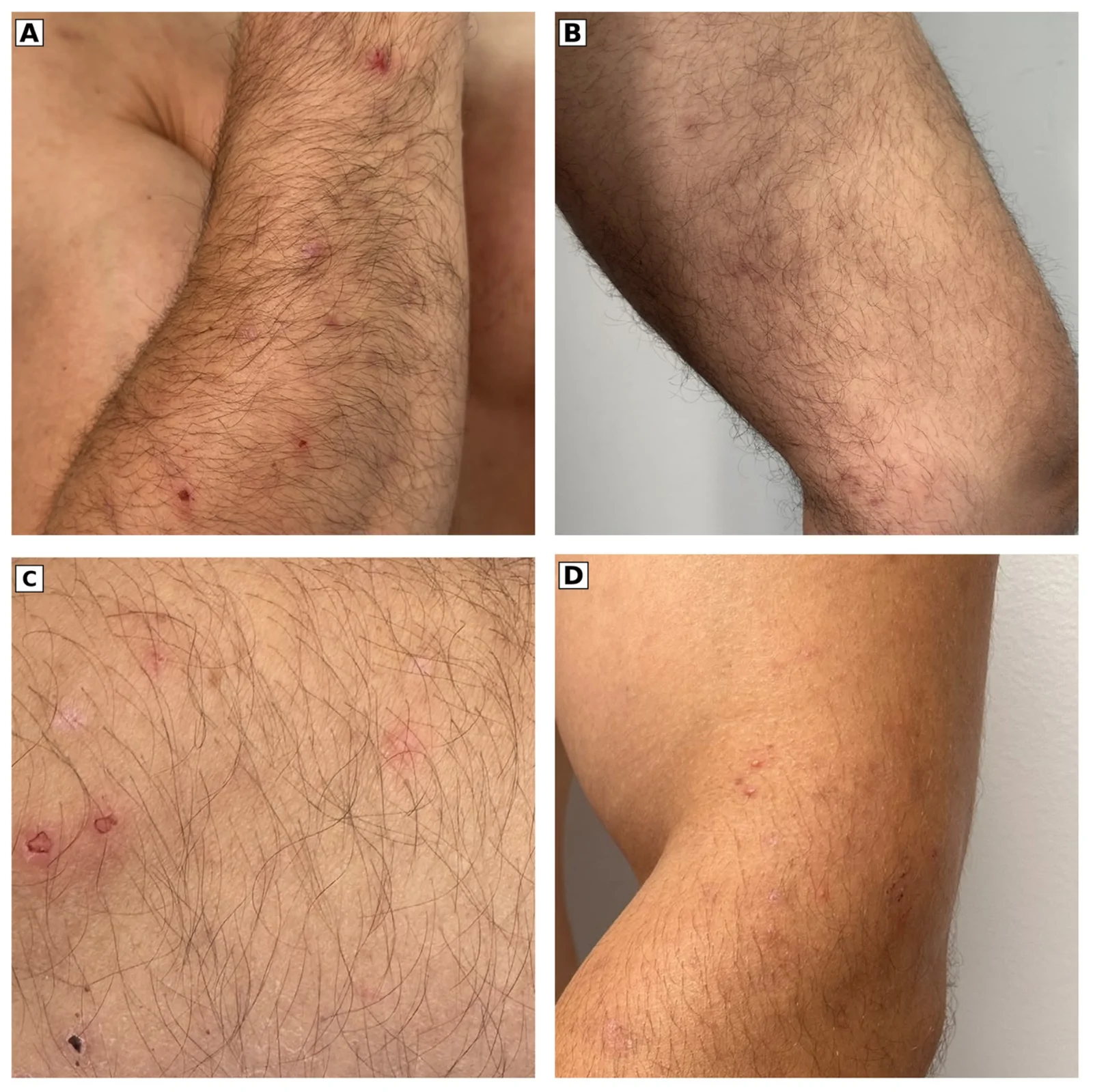
Clinical course of the limb-predominant eruption. (**A**) Medial aspect of the arm and (**B**) thigh and knee at the month 9 flare, before initiation of systemic therapy: excoriated and crusted erythematous papules with post-inflammatory macules. (**C**) Forearm at month 10, three weeks after starting acitretin: persistent excoriated papules. (**D**) Left arm at month 12, three months after starting systemic treatment: residual post-inflammatory macules and a few excoriated papules. Photographs were obtained under routine clinical conditions and are not standardised.

**Figure 2:**
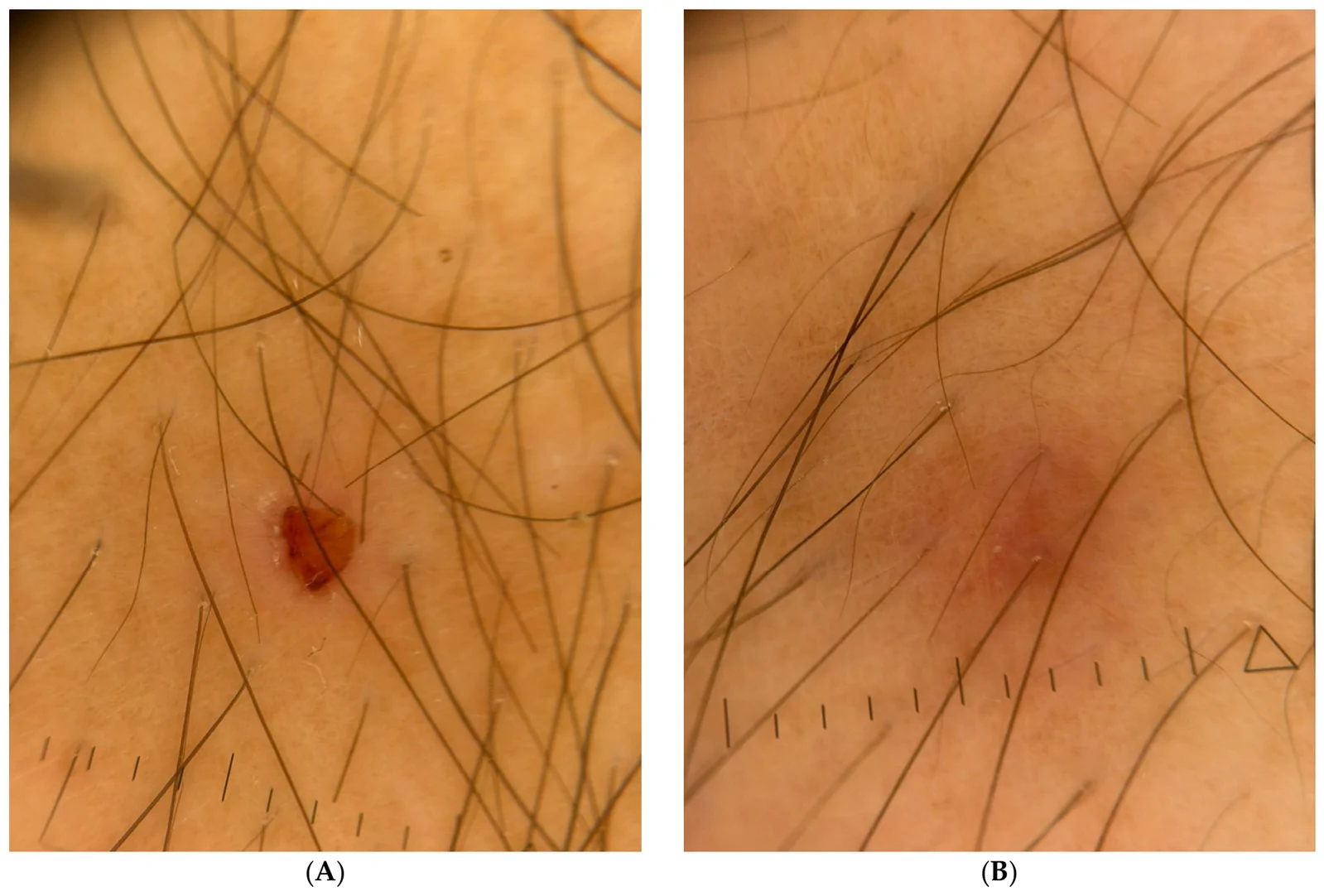
Dermoscopy at the month 9 flare (5× magnification). (**A**) Crusted papule; (**B**) erythema with fine superficial scale. No burrows or delta-wing jet signs were seen.

At the month 9 flare visit, before initiation of systemic therapy, multiple erythematous and erythemato-brown papules, many excoriated with superficial haemorrhagic crusts, were distributed predominantly over the proximal upper limbs and thighs, with only discrete truncal involvement and post-inflammatory macules on the posterior trunk (Figure 1A,B). Vesicles, bullae and pustules were absent. Dermoscopy showed a nonspecific inflammatory pattern of focal pink-red erythema, fine superficial scale and superficial haemorrhagic crusts (Figure 2). No burrows and no delta-wing jet signs were identified at any of the examined sites, and there was no involvement of the interdigital web spaces or the genital skin, findings that argued against scabies.

Routine investigations (complete blood count, erythrocyte sedimentation rate, fibrinogen, glucose, liver enzymes, lipid profile, urea, creatinine, serum ferritin and urinalysis) were largely unremarkable; in particular, aspartate aminotransferase (25 U/L), alanine aminotransferase (19 U/L), gamma-glutamyl transferase (17 U/L) and the lipid profile (total cholesterol 146 mg/dL, low-density lipoprotein (LDL) cholesterol 80 mg/dL, high-density lipoprotein (HDL) cholesterol 58 mg/dL) were within the reference range, and there was no peripheral eosinophilia (eosinophils 0.14 × 10^9^/L, 1.8%). Derived haematological indices commonly used as systemic inflammatory markers were likewise unremarkable (neutrophil-to-lymphocyte ratio 1.28; platelet-to-lymphocyte ratio 83.4) [6]. Lactate dehydrogenase was mildly elevated at 252 U/L (reference 125–220 U/L). Serum creatinine was 1.18 mg/dL (reference 0.6–1.2 mg/dL), and the laboratory reported an estimated glomerular filtration rate (eGFR) of 61.2 mL/min/1.73 m^2^ calculated with the Modification of Diet in Renal Disease (MDRD) equation; urinalysis, including urinary albumin, was normal and there was no clinical evidence of renal disease. This value was interpreted with caution rather than dismissed. The MDRD equation is known to underestimate the glomerular filtration rate in individuals with normal or near-normal kidney function, and recalculation of the same creatinine value with the 2021 Chronic Kidney Disease Epidemiology Collaboration (CKD-EPI) creatinine equation [7] gives an eGFR of approximately 86 mL/min/1.73 m^2^, which, in the absence of albuminuria or any other marker of kidney damage, does not meet criteria for chronic kidney disease; high muscle mass, which raises creatinine-based estimates, is a further plausible contributor. Nevertheless, creatinine and eGFR were not re-measured within the follow-up period reported here, which we acknowledge as a limitation (Section 3.6). Total serum immunoglobulin E (IgE) was moderately elevated at 223.2 IU/mL (reference < 100 IU/mL); allergen-specific IgE testing, lesional cytokine analysis and immunohistochemistry were not performed, and this isolated value was regarded as a nonspecific finding.

**Figure 3:**
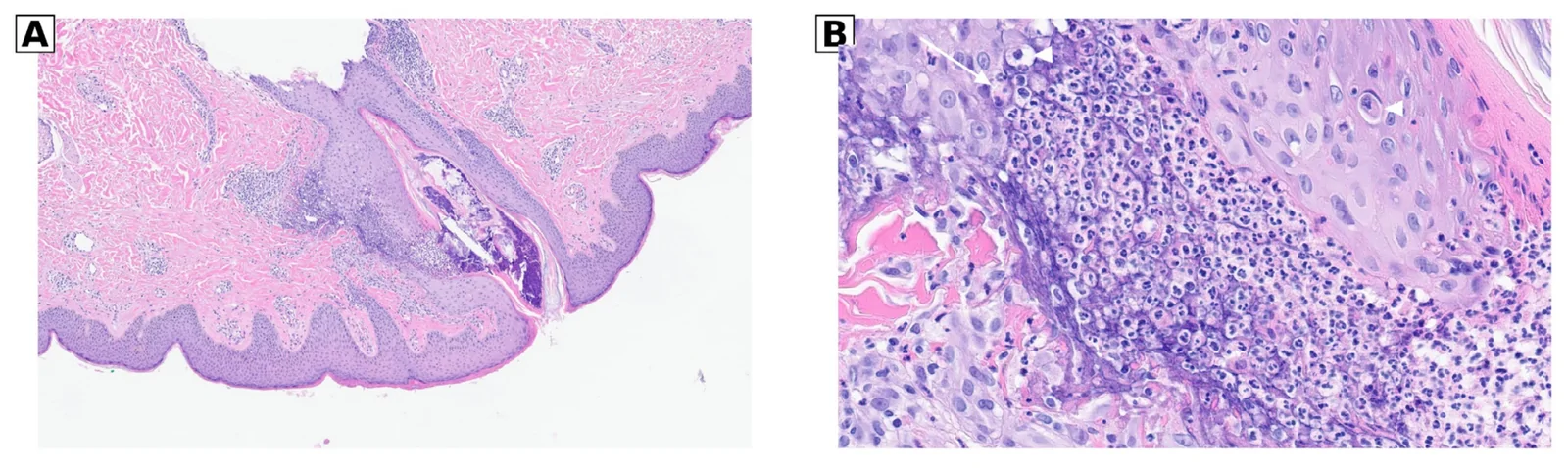
Histopathology of the shave biopsies from the right arm and forearm at the month 9 flare (haematoxylin and eosin). (**A**) Spongiotic-acanthotic epidermis with focal parakeratosis and a mixed lichenoid, perifollicular and perivascular inflammatory infiltrate (original magnification 4×). (**B**) Focus of acantholysis (arrow) at the margin of a fibrinoneutrophilic exudate; dyskeratotic keratinocytes are indicated by arrowheads (original magnification 40×).

At the month 9 flare, two shave biopsies were obtained from active papules on the right arm and the right forearm before treatment was initiated. Both showed similar changes, consisting of a spongiotic-acanthotic epidermis with focal non-confluent parakeratosis, a mixed inflammatory infiltrate with a lichenoid, perifollicular and perivascular distribution, small foci of acantholysis with occasional dyskeratotic keratinocytes, associated with a fibrinoneutrophilic exudate (Figure 3). Although acantholysis was focal, its recurrence in specimens obtained during a separate disease episode, in the same clinical setting as the initial biopsy, was regarded as the key finding supporting the diagnosis; the coexisting spongiotic and lichenoid features are recognised histological patterns of GD rather than arguments against it (Section 3.3).

On the basis of clinicopathological correlation, a diagnosis of chronic-recurrent GD with an atypical, limb-predominant distribution was made and coded as L11.1 in the International Classification of Diseases, 10th Revision (ICD-10). The diagnosis rested on the following elements: a recurrent, intensely pruritic eruption of discrete, non-follicular, non-grouped papules precipitated by occupational heat and exertion; the repeated demonstration, in biopsies from two separate disease episodes, of focal acantholysis accompanied by dyskeratosis, within an otherwise spongiotic and lichenoid background; the absence of the confluent suprabasal or full-thickness acantholysis, the numerous corps ronds and grains, and the seborrhoeic or flexural distribution characteristic of Darier and Hailey–Hailey disease, together with a negative family history; and the absence of clinical or immunofluorescence evidence of pemphigus. Hereditary acantholytic dermatoses, pemphigus, scabies, folliculitis, early pityriasis rubra pilaris, papular eczema and arthropod reactions were considered (Table 2); none was supported by the clinical, histological or immunofluorescence findings, with the caveats regarding immunofluorescence discussed in Section 3.3 and Section 3.6. Treatment was started at the month 9 visit with acitretin 20 mg/day (body weight 87 kg, corresponding to 0.23 mg/kg/day) for 2 weeks, followed by 10 mg/day (0.11 mg/kg/day) as a planned step-down in response to the early clinical improvement, fexofenadine 180 mg/day, and clobetasol propionate 0.05% applied to active lesions during flares, together with barrier measures (short lukewarm showers, a fragrance-free syndet, a daily reparative emollient, loose cotton clothing and avoidance of overheating). Liver enzymes, lipid profile and renal function were assessed at baseline, and the patient was reassessed clinically at 3 weeks and 3 months. Dupilumab, which remains off-label for GD, was not initiated. Seasonal monitoring was planned so that any further flare could be detected early.

**Figure 4:**
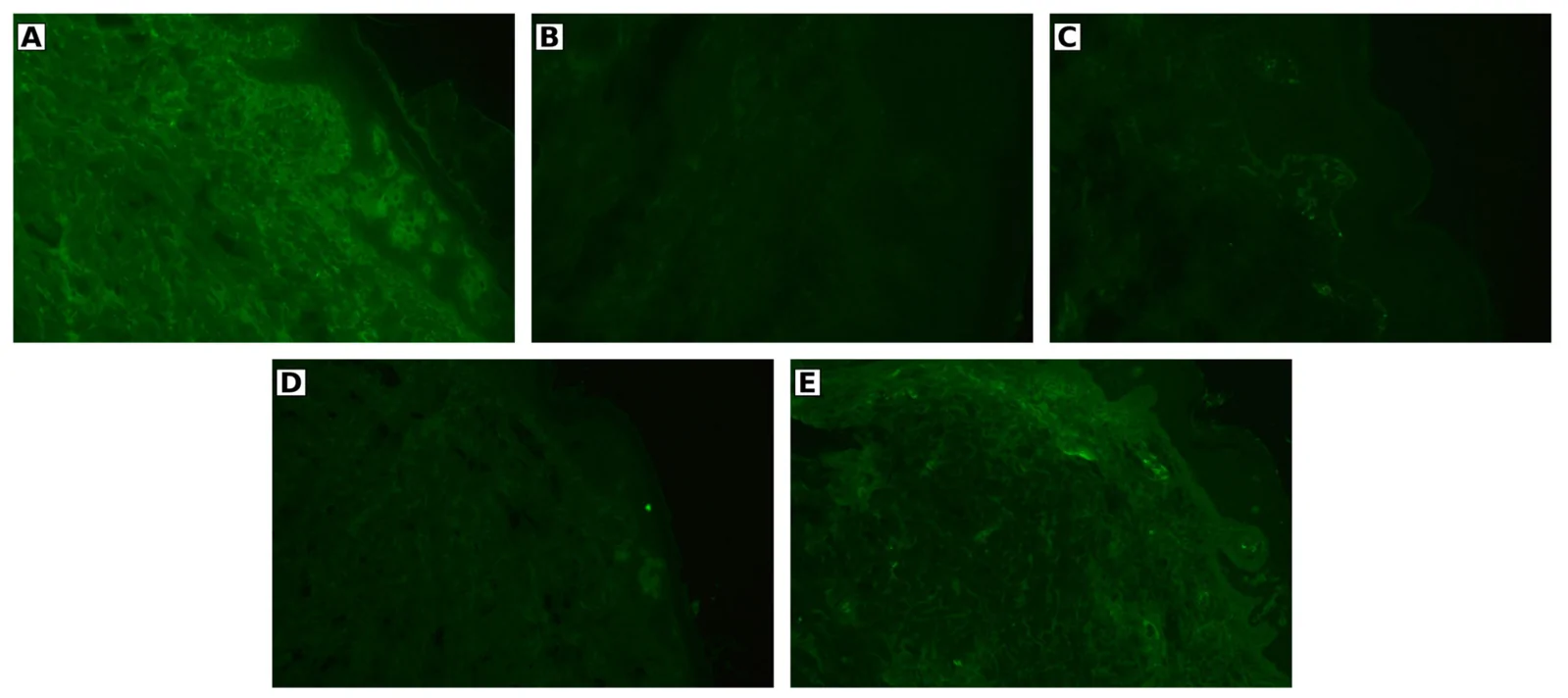
Direct immunofluorescence of a punch biopsy from lesional skin obtained at month 10, during topical clobetasol therapy (original magnification 10×). (**A**) Granular IgG deposition along the basement-membrane zone; (**B**) IgA negative; (**C**) weak focal granular IgM deposition along the basement-membrane zone; (**D**) C3c negative; (**E**) fibrin 1+. The pattern was considered nonspecific; it does not support, but cannot by itself exclude, an autoimmune blistering disorder (Section 3.6).

At the month 10 visit, three weeks after acitretin had been started, partial regression of the initial papular lesions was observed, but pruritus persisted and new papular lesions had developed on the abdomen, a previously spared site, and on the upper and lower extremities (Figure 1C), indicating ongoing disease activity. Because new lesions had appeared at a previously uninvolved site despite treatment, and because acantholysis had been only focal in the earlier specimens, the purpose of this visit was to re-examine the diagnosis rather than to assess treatment response: punch biopsies were obtained from the upper limb, one from the arm and one from the forearm, for histopathology and for direct immunofluorescence, which had not been performed previously and was carried out on lesional skin. The sampled skin had been exposed to topical clobetasol for up to three weeks; the new abdominal lesions, which had not been treated topically, were not biopsied (Section 3.6). Histological examination showed an acanthotic, hypogranular epidermis with focal parakeratosis and a predominantly neutrophilic inflammatory exudate; no acantholytic foci were identified in the sections examined. Direct immunofluorescence (DIF) showed negative immunoglobulin A (IgA) and complement C3c, weak focal granular immunoglobulin M (IgM) deposition along the basement-membrane zone, granular immunoglobulin G (IgG) deposition at the basement-membrane zone, and fibrin 1+ (Figure 4). These findings were considered nonspecific: the absence of intercellular IgG and C3 deposits did not support pemphigus, and the granular basement-membrane deposits were not accompanied by any clinical or histological feature of a subepidermal autoimmune blistering disease or of lupus erythematosus. Two limitations apply to this result. The biopsy was obtained from lesional rather than perilesional skin and under potent topical corticosteroid therapy, both of which reduce the sensitivity of DIF, and a serum autoimmune profile (indirect immunofluorescence or enzyme-linked immunosorbent assays for antibodies against desmoglein 1 and 3 and against the bullous pemphigoid antigens BP180 and BP230) was not obtained; the DIF result therefore lowers the probability of an autoimmune blistering disorder without formally excluding it. The absence of acantholysis in these sections was regarded as plausibly attributable to the histological modification of lesions treated with a potent topical corticosteroid, compounded by the focal nature of the process, rather than as evidence against the diagnosis; taken together with the two previous biopsies demonstrating focal acantholysis, the clinicopathological picture remained consistent with GD, and treatment was continued unchanged.

At the month 12 visit, approximately three months after acitretin had been started, the eruption had improved markedly. The trunk, the thighs and the legs had cleared, and the upper limbs had improved substantially, with only residual post-inflammatory macules and a few excoriated papules persisting on the forearms (Figure 1D); no new vesicles or pustules had appeared. On dermoscopy the previously described pink-red erythema had faded and no active papules or superficial crusts were identified at the sites examined. The DLQI had fallen from 17/30 at the month 9 flare to 5/30. Treatment was taken as prescribed, and no adverse effects were reported at any visit. Liver enzymes and lipid profile were within the reference range at baseline; in the absence of symptoms or adverse effects, laboratory monitoring was not repeated during the three-month follow-up, which we acknowledge as a limitation. No objective lesion count, body surface area estimate or numerical pruritus rating had been recorded prospectively, so the outcome assessment relies on clinical photographs obtained under routine conditions and on the DLQI. Because acitretin, the antihistamine, the topical corticosteroid and barrier care were started together, and because GD follows a naturally fluctuating course, the improvement can be described only as temporally associated with the combined regimen and cannot be attributed to acitretin specifically. Acitretin was discontinued after three months of treatment. At the last follow-up contact the patient remained free of new skin lesions, with disease control maintained through consistent barrier-directed care alone (regular emollients, avoidance of heat exposure and avoidance of tight clothing).

## 3. Discussion

The literature underpinning this discussion was identified by searching Scopus (TITLE-ABS-KEY), the Web of Science Core Collection (Topic) and PubMed (Title/Abstract) on 12 August 2026, without language or date restrictions, using the terms “Grover disease”, “Grover’s disease”, “transient acantholytic dermatosis” and “persistent acantholytic dermatosis”. The searches returned 304, 330 and 317 records, respectively. Records were screened for relevance and supplemented by citation tracking of the retrieved articles. This is a narrative rather than a systematic review; no formal eligibility criteria, duplicate screening or risk-of-bias assessment were applied, and the selection of cited works therefore reflects relevance to the questions raised by the present case, namely the histopathological patterns and diagnostic criteria of GD, its atypical presentations (young age, extra-truncal distribution and chronic-recurrent course), the value and limitations of direct immunofluorescence and dermoscopy, current management, and the emerging molecular and immunological context.

### 3.1. Epidemiology and Clinical Spectrum

GD predominates in men older than 50 years and is a frequent incidental diagnosis among older and hospitalised adults, in whom heat, sweating and prolonged bed rest are common contributors [1,2]. Presentation in younger individuals is uncommon and easily overlooked; several reported young patients have been women or adolescents [8,9]. The clinical spectrum is broad. Beyond the classic discrete, keratotic, pruritic papules and papulovesicles of the trunk, GD may follow Blaschko lines as segmental disease [10], present with oedematous erythema or other atypical morphologies [11,12], be confounded by concurrent infestation [13], or assume a bullous appearance [9,14]. Crucially, the label “transient” is frequently inaccurate: a substantial subset pursues a persistent or chronic-recurrent course over months to years, sometimes with seasonal periodicity tied to heat exposure [15]. Anatomically, the central trunk is the site of predilection and the proximal extremities are the next most frequently involved region in standard descriptions; predominantly limb-based disease with only discrete truncal involvement, as in our patient, is described but remains uncommon [1,2]. Our patient combines several atypical features, namely young age, a largely extra-truncal, limb-predominant distribution involving the proximal upper limbs and thighs, and a chronic-recurrent course with flares triggered by occupational heat and exertion, a constellation that can mislead towards eczema, folliculitis or arthropod reactions and delay diagnosis. Each of these features has been reported individually, and a chronic-recurrent course is in fact common; their combination in a single patient is less often documented but should not be regarded as exceptional. GD should therefore remain in the differential of persistent pruritic papular eruptions even when the patient is young and the distribution is atypical.

### 3.2. Triggers and Disease Associations

The canonical triggers of GD (heat, sweating, ultraviolet exposure, xerosis, occlusion and prolonged bed rest) reflect a tendency for disease to surface when the epidermis is thermally or mechanically stressed, and unusual precipitants continue to be reported [1,16]. Drug-induced GD is an expanding category, implicating cytotoxic chemotherapy such as taxanes [17,18], aromatase inhibitors [19], and biologic or bispecific agents [20,21]. The most clinically salient association is with immune checkpoint inhibitor (ICI) therapy: acantholytic dermatoses, including GD, are recognised cutaneous immune-related adverse events, whose epidemiology and management have been characterised at the population and review level [22,23,24], and which can be supported diagnostically by reflectance confocal microscopy [25]. GD may co-occur with bullous pemphigoid under anti-programmed cell death protein 1 (PD-1) blockade, suggesting shared immunologic mechanisms [26,27,28], while cutaneous immune-related toxicity has itself been associated with improved oncologic survival [29]. Such immunotherapy-related, refractory disease has been reported to respond to dupilumab [30]. GD-like eruptions have also been reported following severe acute respiratory syndrome coronavirus 2 (SARS-CoV-2) infection and vaccination, broadening the list of immunologic triggers [31,32,33]. Finally, GD has been linked to keratinocyte carcinomas and internal malignancy; although causality remains debated, this association argues for vigilance in older or recalcitrant patients [34,35,36]. In our young patient without additional oncologic risk factors, no disease-specific malignancy screening beyond routine age- and risk-appropriate care was considered necessary.

### 3.3. Histopathology, Dermoscopy, and Direct Immunofluorescence

Histologically, GD is best regarded as a reaction pattern rather than a single entity. The four classic acantholytic patterns, Darier-like (suprabasal acantholysis with dyskeratosis), Hailey–Hailey-like (broader acantholysis), pemphigus vulgaris-like and pemphigus foliaceus-like, are complemented by the spongiotic pattern and, since the reappraisal of 120 cases by Fernández-Figueras et al., by lichenoid, vesicular, porokeratotic, lentiginous and dysmaturative patterns, so that at least ten histological patterns are now recognised, and more than one frequently coexists in a single specimen [1,37,38]. Two consequences follow for diagnosis. First, acantholysis is characteristically focal and may be minimal or absent in a given section, so that a biopsy without acantholysis does not exclude the diagnosis and additional levels, or a further biopsy from an active, untreated lesion, may be required. Second, spongiotic and lichenoid changes, which dominated the specimens from our patient, are themselves part of the histological spectrum of GD and should not, in the presence of focal acantholysis and a compatible clinical picture, redirect the diagnosis towards eczema or a lichenoid dermatitis. As in other settings in which the clinical picture alone is insufficient for diagnostic certainty, histopathological examination was decisive in this case [39]. In our patient, focal acantholysis was documented both in the initial biopsy and in the two specimens obtained during the month 9 flare, whereas in the specimens obtained at month 10, after up to three weeks of potent topical corticosteroid therapy, acantholysis was not identified in the sections examined and the changes consisted of acanthosis, hypogranulosis, focal parakeratosis and a predominantly neutrophilic exudate. We regard this difference as plausibly attributable to the histological modification of treated lesions, compounded by the sampling variability inherent to a focal process, rather than as evidence of intrinsic disease heterogeneity. Ancillary immunohistochemical markers such as claudin-4 and IgG4 have been explored to refine the diagnosis of acantholytic and bullous disorders [40,41], and interleukin-13 (IL-13) immunohistochemistry has been applied across type 2-associated dermatoses, although GD showed only minimal IL-13 protein expression in that series [42]. Direct immunofluorescence is characteristically negative or nonspecific in GD and is used principally to evaluate for pemphigus and other immunobullous diseases [1]; its sensitivity is reduced when the biopsy is obtained under topical or systemic corticosteroid therapy, as in our patient, so that a nonspecific result lowers the probability of an autoimmune blistering disorder without excluding it, particularly when serum autoantibody testing is not available. Dermoscopy is likewise nonspecific, typically showing erythema, fine scale and crust, and serves mainly to exclude alternative diagnoses such as scabies [43], although ultraviolet-induced fluorescence and sub-ultraviolet reflectance dermatoscopy have recently been proposed to add diagnostic information [44]. Awareness of GD-like patterns in early pityriasis rubra pilaris and of papular acantholytic dermatosis of genital skin helps avoid misclassification [45,46].

### 3.4. Molecular and Immunological Context

The molecular understanding of GD now frames the disease as a disorder of keratinocyte adhesion. Somatic damaging variants in ATP2A2, the gene mutated in the germline in Darier disease, have been identified in lesional GD skin [3]. ATP2A2 encodes the sarco/endoplasmic reticulum calcium ATPase isoform 2 (SERCA2), whose dysfunction perturbs the calcium signalling on which desmosomal assembly depends, and hyperactivation of the ERK/mitogen-activated protein kinase (MAPK) pathway in keratinocytes impairs intercellular adhesion and reproduces a GD-like phenotype [4,47]; transcriptional profiling has revealed gene-expression programmes shared across Darier disease, Hailey–Hailey disease and GD [48]. Superimposed on this adhesion defect, transcriptomic analysis of 31 lesional biopsies identified increased interleukin-13 expression as a hallmark of GD, and nine treatment-refractory patients in a supportive series improved with dupilumab [5]; IL-13 protein, however, was only minimally detectable by immunohistochemistry in GD [42], so that tissue-level evidence for type 2 involvement currently rests on transcript rather than protein data. None of these mechanisms was investigated in our patient: ATP2A2 sequencing, lesional cytokine analysis and immunohistochemistry were not performed, and they are presented here strictly as emerging mechanisms reported in the literature. The only patient-specific finding that could be discussed in this context is a single moderately elevated total serum IgE (223.2 IU/mL), obtained without peripheral eosinophilia and without allergen-specific IgE testing. Total serum IgE is a nonspecific marker that is elevated in a range of unrelated conditions and in a proportion of healthy adults; it cannot establish a type 2 immune phenotype in an individual patient, predict response to interleukin-4 (IL-4)/IL-13 blockade or serve as a diagnostic marker of GD, and we report it as an incidental laboratory observation. Whether serum IgE or other accessible markers have any prognostic or predictive value in GD would require prospective evaluation in adequately sized cohorts.

### 3.5. Management

Treatment of GD is largely empirical and proceeds in a stepwise fashion (Table 3). First-line measures address triggers and barrier function: avoidance of heat, sweating, ultraviolet exposure and friction; emollients and barrier repair; and topical corticosteroids, with topical calcineurin inhibitors as steroid-sparing options [1]. Antihistamines provide symptomatic relief of pruritus [1]. For disease refractory to topical therapy, systemic retinoids (acitretin, isotretinoin) are a common first systemic choice and have been used in complex settings such as post-transplantation and graft-versus-host disease [49,50], while methotrexate has shown efficacy comparable to acitretin in topical-refractory disease in a retrospective comparison [51]. Phototherapy yields mixed results and carries the paradox that ultraviolet light is itself a recognised trigger; blue-light phototherapy has been evaluated in a nonrandomised controlled trial [52]. Dupilumab, an interleukin-4 receptor-alpha (IL-4Rα) antagonist, has been reported to produce rapid and often durable improvement in recalcitrant disease, including immunotherapy-related GD, in case reports, small series and one retrospective study (Table 4) [5,30,53,54,55,56,57,58], and its off-label use across acantholytic dyskeratotic disorders has been summarised [59]. This evidence is uncontrolled and subject to publication bias, and no randomised comparison with retinoids or other systemic agents exists. In our patient, acitretin was chosen as the first systemic agent in line with the stepwise approach summarised in Table 3; dupilumab, which remains off-label for GD, was not initiated and would be considered only if the disease proved refractory to conventional systemic therapy. As discussed in Section 2, the improvement observed under the combined regimen cannot be attributed to any single component. Comprehensive case-based reviews provide practical frameworks for navigating this therapeutic ladder [60].

### 3.6. Strengths, Limitations, and Future Directions

This report has the inherent limitations of a single-case observation. The exact date of disease onset was reconstructed retrospectively, and no validated numerical pruritus score, lesion count or body surface area estimate was recorded prospectively, so that outcome assessment relies on the DLQI and on non-standardised clinical photographs. Several diagnostic limitations deserve emphasis. Direct immunofluorescence was performed on a single occasion, on lesional rather than perilesional skin, exposed to potent topical corticosteroid therapy for up to three weeks, two circumstances that reduce its sensitivity and increase the rate of false-negative results, and a serum autoimmune profile (indirect immunofluorescence, anti-desmoglein 1 and 3, anti-BP180 and anti-BP230 antibodies) and antinuclear antibody testing were not performed. The nonspecific DIF result therefore reduces, but does not eliminate, the probability of an autoimmune blistering disorder; the granular IgG and IgM deposits with fibrin along the basement-membrane zone were interpreted as nonspecific in the absence of clinical or histopathological features of connective tissue disease or of a subepidermal blistering disease. Acantholysis was focal in the diagnostic specimens and absent in the specimens obtained under treatment, which were taken from treated limb skin rather than from the newly involved, untreated abdominal skin, so that sampling variability cannot be excluded, and molecular analysis of lesional skin, including ATP2A2 sequencing, was not performed. The moderately elevated total serum IgE was a single measurement, without allergen-specific IgE, longitudinal values or stool parasitology, and carries no diagnostic or predictive weight. Finally, because several treatments were started simultaneously in a disease with a fluctuating natural course, the observed improvement cannot be attributed to acitretin. Laboratory monitoring during acitretin therapy was limited to the baseline assessment, because the patient remained asymptomatic and free of adverse effects, and the laboratory-reported eGFR was not re-measured within the follow-up period reported here, although recalculation with the 2021 CKD-EPI equation indicates a value of approximately 86 mL/min/1.73 m^2^. The strengths of the report are serial clinicopathological assessment with biopsies at three time points, direct immunofluorescence, dermoscopic assessment arguing against scabies, quality-of-life measurement before and after treatment, and detailed documentation of an atypical age and an atypical, limb-predominant distribution. Future work should establish, through controlled studies, the place of dupilumab relative to retinoids in the treatment algorithm, and should clarify whether type 2 markers or somatic ATP2A2 and ERK-pathway alterations contribute to sporadic and atypical disease.

### 3.7. Barrier-Directed Adjuvant Care: An Unmet Research Need

At present, no dermato-cosmetic product has been specifically developed or clinically evaluated for GD. The adjuvant topical options currently used in practice are supported only by indirect evidence extrapolated from atopic dermatitis, xerosis cutis, chronic pruritus, epidermal barrier repair and post-procedural skin recovery, rather than by dedicated trials in GD (Table 5). Many of the ingredient classes in routine use, including ceramide-, urea- and panthenol-based actives and lamellar lipid vehicles, are mechanistically attractive for a disease characterised by epidermal barrier dysfunction and pruritus; their benefit in GD, however, remains inferential. This gap defines a concrete research direction. Because epidermal barrier dysfunction and pruritus are clinically relevant features of GD, adjuvant topical care deserves to be investigated specifically in this condition rather than inferred exclusively from evidence in atopic dermatitis. What is needed is not another extrapolation but a purpose-designed product evaluated prospectively in GD itself, with barrier and pruritus endpoints, which could in time provide a steroid-sparing adjuvant for chronic-recurrent disease.

The table is organised by ingredient class rather than by commercial product; no brand names or proprietary technology names appear in the table, and nothing in the table constitutes a recommendation of a specific product. No ingredient class listed has been evaluated in a clinical trial specific to GD; the available evidence is indirect and derives from atopic dermatitis, xerosis, chronic pruritus, barrier repair and post-procedural studies. Several, though not all, of the studies cited in support of these classes were funded by, or conducted in collaboration with, the manufacturers of the corresponding products, and should be interpreted accordingly. The authors have no financial or commercial relationship with any manufacturer of the products from which these classes are derived.

This gap is also visible at the level of the published literature. A term co-occurrence map of the titles and abstracts of the PubMed records on GD from the preceding five years, complemented by the references cited in this article (35 records; VOSviewer version 1.6.20 [71]; methods in Supplementary Text S1 and full map in Supplementary Figure S1), shows that terms describing extra-truncal distribution (extremity) are peripheral and infrequent relative to trunk, and that barrier-directed and adjuvant topical care is represented only by isolated low-frequency terms such as emollient, without a dedicated thematic cluster. Within the usual caveats of descriptive science mapping, which depends on indexing coverage and on title and abstract terminology, this supports the view that both the limb-predominant phenotype and adjuvant barrier care remain underrepresented in the GD literature.

## 4. Conclusions

GD should not be regarded exclusively as a transient truncal dermatosis of older men. This case documents a chronic-recurrent, predominantly limb-based phenotype in a young adult in which the diagnosis, although uncommon in this combination of features, was established through clinicopathological correlation across serial biopsies obtained during separate disease episodes. Focal or absent acantholysis in individual specimens, particularly those obtained during potent topical corticosteroid treatment, and the limited value of direct immunofluorescence performed under treatment are practical diagnostic pitfalls illustrated by this case; recognising spongiotic and lichenoid changes as part of the histological spectrum of GD is essential to avoid redirecting the diagnosis towards eczema. The moderately elevated total serum IgE is a nonspecific, incidental finding. Management remains stepwise and individualised; in the present patient, a combined regimen of acitretin, an oral antihistamine, a topical corticosteroid and barrier-directed care was temporally associated with marked clinical improvement, although the contribution of each component and of the natural course of the disease cannot be separated.

## Figures and Tables

**Table 1:** Documented clinical course of chronic-recurrent GD. Time points are expressed as months from the first documented dermatologic assessment (month 0).

Time Point	Symptoms and Clinical Status	Histopathology and Investigations	Treatment	Clinical Response
Month −6 (estimated onset)	Onset reconstructed from the history taken at month 0	Not performed or documented	Not reliably documented	Not reliably documented
Month 0 (first documented assessment)	Intensely pruritic erythematous papules, predominantly on the extremities	Punch biopsies (arm, forearm): focal dyskeratosis and minimal suprabasal acantholysis with spongiosis and a superficial mixed infiltrate; compatible with GD	Symptomatic and topical treatment	Clinical improvement
Month 2	Intermittent moderate pruritus	No new investigations	Bilastine 20 mg/day; topical and barrier-directed care; vitamin D and zinc supplementation prescribed elsewhere (indication not documented)	Intermittently symptomatic
Month 9 (flare)	Limb-predominant papular flare; DLQI 17/30	Two shave biopsies (right arm, right forearm): spongiotic-acanthotic pattern with lichenoid and perivascular infiltrate and small foci of acantholysis with occasional dyskeratotic keratinocytes; compatible with GD	Acitretin 20 mg/day for 2 weeks, then 10 mg/day; fexofenadine 180 mg/day; topical clobetasol during flares; barrier care and trigger avoidance	Systemic treatment initiated
Month 10 (3 weeks after starting acitretin)	Partial regression; persistent pruritus; new papules on the abdomen and limbs	Punch biopsies (arm, forearm) under topical clobetasol: acanthosis, hypogranulosis, focal parakeratosis, neutrophilic exudate; no acantholysis in the sections examined. DIF nonspecific (see text)	Continued systemic and topical treatment	Partial response with ongoing disease activity
Month 12 (3 months after starting acitretin)	Marked improvement; residual macules and a few excoriated papules on the forearms; DLQI 5/30	Clinical and dermoscopic reassessment only	Acitretin discontinued after 3 months; barrier care continued	Marked clinical improvement

DIF, direct immunofluorescence; DLQI, Dermatology Life Quality Index; GD, Grover disease. The approximate onset was reconstructed retrospectively from the history documented at month 0. A validated numerical pruritus score, lesion count or body surface area estimate was not prospectively recorded.

**Table 2:** Differential diagnoses considered, and the features that argued against each of them in the present patient.

Entity	Distinguishing Clinical/Histologic/Immunofluorescence Features
Darier disease	Hereditary (germline ATP2A2); seborrhoeic distribution; persistent; nail and mucosal signs; prominent suprabasal acantholytic dyskeratosis (corps ronds, grains). Not supported: no family history, limb-predominant non-seborrhoeic distribution, no nail or mucosal signs, only focal acantholysis.
Hailey–Hailey disease	Hereditary (ATP2C1); flexural macerated plaques; full-thickness (“dilapidated brick wall”) acantholysis. Not supported: no family history, no flexural plaques, no full-thickness acantholysis.
Pemphigus (foliaceus/vulgaris)	Autoimmune; flaccid bullae/erosions; intercellular IgG/C3 on direct immunofluorescence. Not supported: no bullae or erosions; no intercellular deposits on DIF. Caveat: DIF performed on lesional skin under topical corticosteroid therapy; serum autoantibody testing not done.
Scabies	Burrows in web spaces/genitalia; nocturnal pruritus; mites/eggs on microscopy. Not supported: no burrows or delta-wing jet signs on dermoscopy; web spaces and genital skin uninvolved.
Folliculitis	Follicular papulopustules; neutrophilic folliculitis; organisms on culture. Not supported: papules non-follicular and non-pustular.
Pityriasis rubra pilaris (early)	May show Grover-like acantholysis early; follicular papules, orange-red plaques, islands of sparing. Not supported: no follicular keratotic papules, orange-red plaques or islands of sparing during follow-up.
Papular eczema	Eczematous, ill-defined papules; spongiosis without true acantholysis; often an atopic background; responds to topical anti-inflammatory therapy. Not supported: focal acantholysis with dyskeratosis in two separate episodes; discrete papules; no atopic history.
Papular urticaria/arthropod reaction	Pruritic papules at exposed or bite sites; seasonal/outdoor exposure; superficial and deep perivascular infiltrate with eosinophils and no acantholysis. Not supported: acantholysis present; no eosinophil-rich deep infiltrate.

DIF, direct immunofluorescence; IgG, immunoglobulin G.

**Table 3:** Stepwise therapeutic options for GD.

Therapy	Comment/Evidence	Ref.
Trigger avoidance, emollients, barrier care	First-line; reduce heat, sweating, ultraviolet exposure, friction, overheating	[1]
Topical corticosteroids ± calcineurin inhibitors	First-line for limited disease	[1]
Antihistamines	Symptomatic pruritus control	[1,5]
Systemic retinoids (acitretin, isotretinoin)	Common first systemic option; effective post-transplant/GVHD	[49,50]
Methotrexate	Reported efficacy comparable to acitretin in a retrospective comparison of topical-refractory disease	[51]
Phototherapy (nb-UVB, blue light)	Mixed evidence; ultraviolet may also trigger disease	[52]
Dupilumab (anti–IL-4Rα)	Reported clinical benefit in recalcitrant disease in uncontrolled case reports, small series and one retrospective study (see Table 4)	[5,30,53,54,55,56,57,58,59]
Systemic corticosteroids	Short-term control; frequent relapse	[1,5]

GVHD, graft-versus-host disease; IL-4Rα, interleukin-4 receptor-alpha; nb-UVB, narrow-band ultraviolet B.

**Table 4:** Reported use of dupilumab in GD and related acantholytic dyskeratotic disorders.

**Report (Ref.)**	**Study Design**	**Clinical Context**	Reported Outcome
Butler et al., 2021 [53]	Case report	GD	Early report of benefit; clinical improvement
Barei et al., 2022 [54]	Case report	GD	Improvement; proposed as future option
Shelton et al., 2022 [30]	Case report	Immunotherapy-related refractory TAD	Prompt resolution
Kaprive et al., 2024 [55]	Case report	Resistant GD	Successful treatment
Murphy et al., 2025 [5]	Observational study: cross-sectional transcriptomic analysis of lesional biopsies (*n* = 31) and retrospective case series (*n* = 9)	GD; increased IL-13 expression identified as a transcriptomic hallmark	9/9 refractory patients improved on dupilumab
Amara et al., 2025 [56]	Retrospective observational study (uncontrolled)	Treatment-resistant GD	Reported efficacy, especially with atopic history
Magnano et al., 2025 [57]	Case series (*n* = 2) with literature review	Recalcitrant GD	Sustained long-term response
McCarter et al., 2025 [58]	Case report	Refractory GD	Clinical response
Udupa et al., 2026 [59]	Systematic review	Acantholytic dyskeratotic disorders (incl. GD)	Off-label dupilumab efficacy/tolerability summarised

GD, Grover disease; IL-13, interleukin-13; TAD, transient acantholytic dermatosis. Outcomes are as reported in uncontrolled studies; controlled data are lacking.

**Table 5:** Potential classes of barrier-directed topical adjuncts relevant to GD and the supporting indirect evidence.

Ingredient Class	Representative Actives	Rationale in Barrier-Dependent, Pruritic Skin	Evidence Base	Potential Relevance to GD
Ceramide-dominant emollients and syndets	Ceramides NP/AP/EOP, cholesterol, free fatty acids, hyaluronic acid, glycerin	Restore the intercellular lipid lamellae and reduce transepidermal water loss	Cohort and review data in atopic dermatitis and xerosis [61,62]	Gentle cleansing and barrier repair in a disease in which epidermal barrier dysfunction is clinically relevant
Humectant therapy	Glycerol, hyaluronic acid	Increase stratum corneum hydration and improve the water-holding capacity of the epidermis	Randomised and review data in xerosis and atopic dermatitis [63,64,65]	Reduction of xerosis and of the sensation of skin tightness
Multivesicular and lamellar lipid vehicles	Bilayer emulsions delivering ceramides and cholesterol in a lamellar arrangement	Prolong lipid delivery and hydration compared with conventional emulsions	Review of multivesicular ceramide-containing emulsions [61]	Sustained hydration between applications
Niacinamide-containing emollients	Niacinamide, shea butter, glycerin	Anti-inflammatory and barrier-supporting; reduce discomfort on washing	Emollient studies in atopic dermatitis [62,66]	Reduction of irritation induced by washing and heat
Microbiome-modulating emollients	Postbiotic bacterial lysates and prebiotic sugars	Modify the cutaneous dysbiosis that accompanies barrier failure	Controlled study showing clinical improvement together with modification of the skin microbiota in atopic dermatitis [66]	Pruritus reduction and support of the cutaneous microbiome
Urea-based hydrating and keratolytic preparations	Urea 5–10%, natural moisturising factor components	Bind water within the stratum corneum and reduce fine scaling	Consensus recommendations for xerosis cutis, in which urea is regarded as a reference active [67]	Deep hydration and reduction of scaling and discomfort
Anti-pruritic barrier formulations	Palmitoylethanolamide, menthoxypropanediol, decanediol, licochalcone A	Provide symptomatic anti-pruritic and soothing effects through heterogeneous mechanisms, including sensory-receptor modulation and anti-inflammatory activity	Evidence limited to small manufacturer-reported studies in atopic dermatitis-related pruritus and not independently replicated	Potential adjunctive control of pruritus between emollient applications
Panthenol- and madecassoside-based repair preparations	Panthenol 5%, madecassoside, zinc, copper	Accelerate re-epithelialisation of excoriations and superficial erosions	Clinical studies after fractional CO2 laser and in irritated skin [68,69]	Repair of scratch-induced microlesions
Copper–zinc and sucralfate-type barrier agents	Copper sulfate, zinc sulfate, sucralfate, smectite clays	Astringent, antiseptic and film-forming protection of excoriated skin	Real-world effectiveness and tolerability study in sensitive and irritated skin [70]	Protection of traumatised or fissured areas
Occlusive emollients	Petrolatum, glycerol, liquid paraffin	Reduce transepidermal water loss by occlusion	Randomised controlled trial in children with atopic dermatitis, flare-prevention randomised study and review of basic emollients in xerosis [63,64,65]	Reduction of xerosis and of the itch–scratch cycle
Oat-extract and prebiotic emollients	Colloidal oat extract, prebiotic sugars, vitamin B3	Soothing and barrier-supporting in sensitive, xerotic skin	Indirect evidence from emollient reviews in atopic dermatitis and xerosis [65]	Reduction of scratching in sensitive, xerotic skin

## Data Availability

The data presented in this study are available on request from the corresponding authors due to privacy restrictions.

## Supplementary Materials

The following supporting information can be downloaded at: https://www.mdpi.com/article/10.3390/diagnostics16193176/s1, Text S1: Retrieval and mapping methods for the bibliometric map; Figure S1: Bibliometric map of the GD research landscape (term co-occurrence network generated in VOSviewer).
