# Supplementary material for: Chronic-Recurrent, Limb-Predominant Grover Disease in a Young Adult: A Clinicopathological Diagnostic Challenge
Source: Diagnostics (Basel). 2026 Sep 30;16(19):3176. doi: 10.3390/diagnostics16193176 (PMC13664335; doi:10.3390/diagnostics16193176)
Supplement: Supplementary file 1 [file diagnostics-16-03176-s001.zip › diagnostics-4533766-supplementary.pdf]

Supplementary Materials

Chronic-Recurrent, Limb-Predominant Grover Disease in a Young Adult: A Clinicopathological Diagnostic Challenge

Laura Maghiar, Andreea-Adriana Neamțu, Teodor-Andrei Maghiar, Mariana Ganea, Ovidiu Țică, Ruxandra Florina Bodog, Teodora Maria Bodog, Octavia Gligor, Laura Grațiela Vicaș, Mădălin Florin Ganea, Csaba Nagy and Ilarie Brihan

Text S1. Retrieval and mapping methods

To situate the present case within the wider research context of Grover disease (GD), a term co-occurrence network of the published literature was constructed by science mapping. Bibliographic records were retrieved from PubMed using the query ("Grover disease" OR "Grover's disease" OR "transient acantholytic dermatosis"), restricted to the preceding five years, and were complemented by the references cited in the main article; the resulting corpus comprised 35 records exported in NBIB format. Terms were extracted from titles and abstracts in VOSviewer version 1.6.20 [71] using the full counting method, with a minimum of two occurrences per term; 157 terms met this threshold, of which the 60% most relevant were retained, in line with the program default. Clustering was performed with the default resolution of 1.00 and a minimum cluster size of one. Node size is proportional to the frequency of each term, the distance between nodes and the thickness of the connecting lines reflect co-occurrence strength, and colour denotes cluster membership as assigned by the association-strength normalisation and modularity-based clustering algorithm implemented in VOSviewer.

The map resolved into five main thematic clusters: clinical management, epidemiology and drug safety (red); phenotypic description, triggers and precipitating exposures (green); diagnostic confirmation and laboratory modalities (blue); case-based evidence and literature synthesis (yellow); and acantholysis, molecular pathophysiology and related genodermatoses (purple). Two observations are relevant to the present report. First, terms describing extra-truncal distribution (extremity) remain peripheral and weakly connected relative to trunk, consistent with the relative under-representation of the limb-predominant phenotype in the mapped literature. Second, barrier-directed and adjuvant topical care is represented only by isolated low-frequency terms such as emollient, without a dedicated cluster. These findings are descriptive, depend on the indexing coverage of the bibliographic source and reflect title and abstract terminology rather than the full content of the retrieved articles.

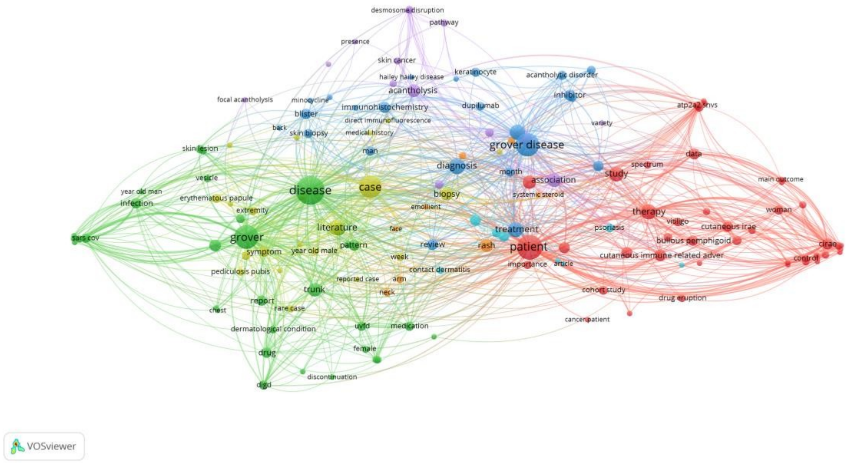

Figure S1. Bibliometric map of the GD research landscape. Term co-occurrence network generated in VOSviewer from the titles and abstracts of the retrieved records (n = 35). Node size is proportional to the frequency of occurrence of each term, the proximity between nodes and the thickness of the links reflect the strength of co-occurrence, and colours indicate the thematic clusters identified by the clustering algorithm.

Reference

71. van Eck, N.J.; Waltman, L. Software survey: VOSviewer, a computer program for bibliometric mapping. *Scientometrics* **2010**, *84*, 523–538. https://doi.org/10.1007/s11192-009-0146-3
